# Did the periodic intensification of routine immunisation strategy (Intensified mission Indradhanush) reduce the demand for pediatric antibiotic formulations in India?

**DOI:** 10.1186/s12879-025-11082-3

**Published:** 2025-05-24

**Authors:** Habib Hasan Farooqui, Anup Karan, Aashna Mehta, Giridhara Rathnaiah Babu, Onno C. P. van Schayck

**Affiliations:** 1 Department of Population Medicine, College of Medicine, QU Health, Qatar University, P.O. Box 2713, Doha, Qatar; 2 Public Health Foundation of India, KIIT Campus, Sohna Road, Gurugram, 122001 India; 3 Care and Public Health Research Institute, Maastricht University, P.O. Box 616, Maastricht, Netherlands

**Keywords:** India, Antibiotic utilisation, Immunisation, Intensified mission Indradhanush, Interrupted time series

## Abstract

**Background:**

Recent research has indicated an increase in antibiotic utilisation, particularly pediatric formulations. Furthermore, an increasing trend in antimicrobial resistance rates has also been reported. Empirical evidence suggests that immunisation reduces the demand for antibiotics. We examined the potential impact of the periodic intensification of the routine immunisation strategy - Intensified Mission Indradhanush (IMI), which was implemented from October 2017 to January 2018, on antibiotic utilisation in India.

**Methods:**

We analysed the PharmaTrac dataset to assess the impact of IMI on antibiotic utilisation. We conducted interrupted time series analyses by fitting a Poisson regression model. We used Newey–West standard errors to account for autocorrelation and heteroskedasticity.

**Findings:**

Poisson segmented regression analysis showed a 12.6% decrease in Fluoroquinolones sales in the first month of IMI implementation (incidence rate ratio [IRR] 0.874, 95%CI: 0.777–0.983). However, post-intervention, their sales remained broadly constant (IRR 1.000, 95%CI 0.995–1.006). Similarly, Chloramphenicol sales decreased by 0.6% in the first month, whereas sales increased by a trend of 0.4% per month (IRR 1.004, 95%CI 0.991–1.017) post-intervention. Interestingly, Trimethoprim sales increased by 17.1% in the first month but decreased by 0.4% per month (IRR 0.959, 95%CI 0.945–0.973) post-intervention. However, there was a modest increase in sales of Macrolides by 3.0%, Cephalosporins by 2.9% and broad-spectrum Penicillin by 0.2% in the first month. Thereafter, Macrolides sales increased by a monthly rate of 0.5% (IRR 1.005, 95% CI 1.000–1.010), Cephalosporins increased by 0.5% (IRR 1.005, 95% CI 1.000 -1.010) and Broad spectrum penicillin increased by 0.9% (IRR 1.009, 95% CI 1.004–1.013) in the post-intervention period. Furthermore, there were heterogeneities across Indian states.

**Interpretation:**

The IMI’s impact on antibiotic utilisation was heterogeneous across antibiotic classes and Indian states.

**Supplementary Information:**

The online version contains supplementary material available at 10.1186/s12879-025-11082-3.

## Introduction

India has one of the highest burdens of childhood infections in the world. As per the latest Global Burden of Disease (GBD) Study 2016 estimates, diarrhoeal diseases and lower respiratory infections continue to remain among the top five leading causes of Disability Adjusted Life Years (DALYs) in India [1]. This high burden of infectious diseases in children is also reflected in antibiotic prescription patterns at the country level. Recent research indicates that in the private sector, the antibiotic prescription rates were highest for children aged 0–4 years and the majority of the antibiotic prescriptions were dispensed for acute upper respiratory infections (20.4%), unspecified acute lower respiratory infections (12.5%), cough (4.7%); and acute nasopharyngitis (4.6%) which do not necessarily require antibiotics [2]. More recent estimates suggest that between 2011 and 2019, the annual antibiotic consumption rate in India decreased by 3.6% [3]. However, there were wide heterogeneities across the Indian States, and inappropriate antibiotic use among High Focus states (Bihar, Chhattisgarh, Jharkhand, Madhya Pradesh, Odisha, Rajasthan, Uttar Pradesh and Northeastern states) increased during the same period. Koya et al. also reported a relative increase of 21.2% for oral liquid preparations that are used in the paediatric population [3]. Though access to medicines is an important concern in India, antimicrobial resistance (AMR) is an emerging threat. Klein et al. reported that in 2019, India had the highest Drug Resistance Index (DRI) rate, a composite index that combines antibiotic consumption rates and resistance across multiple pathogen combinations, and therefore the lowest relative effectiveness of antibiotic therapy [4]. 

Vaccines remain one of the most cost-effective interventions to reduce infectious disease burden at the individual and population levels [5, 6]. Empirical evidence suggests that vaccines not only protect against primary and secondary infections from vaccine-preventable diseases they also reduce the demand for antibiotics [7]. Theoretical [8], empirical [9] and modelling studies [10] have demonstrated that vaccination against a target pathogen directly reduces disease incidence. This entails reduced antibiotic consumption and AMR for that pathogen, and at the same time, reduces the potential for AMR in other pathogens. Hence, estimating the potential impact of vaccination on the reduction of demand for antibiotics is an important policy question.

India’s Universal Immunisation Programme (UIP) offers twelve vaccines free of cost to a cohort of 27 million children and 30 million pregnant women through 12 million sessions annually [11]. More recently, the Ministry of Health and Family Welfare (MOHFW) launched Intensified Mission Indradhanush (IMI)– a periodic intensification of routine immunisation strategy - between October 2017 and January 2018 to address socioeconomic inequalities in access to immunisation to achieve immunization coverage of 90% in districts and urban areas with previously low levels of coverage, across 24 states [12]. The implementation strategy included the identification of under-immunised children by conducting door-to-door surveys and appropriate locations for immunisation sites, with particular emphasis on urban slums and nomadic populations through district-level micro-plans. The increase in full immunization coverage (FIC) after IMI, compared with National Family Health Survey (NFHS-4) was highest for Assam (31% points), followed by Madhya Pradesh (26.4% points), North-Eastern states (23.9% points), Maharashtra (20.3% points), Bihar (15.3% points), Uttar Pradesh (15.2% points) and Rajasthan (11.6% points). The impact of IMI on the improvement of coverage rates of standard UIP vaccines in the implementation districts is well documented [13]. However, the impact of IMI on antibiotic usage isn’t known.

We hypothesized that an increase in immunisation coverage rates under the IMI program should lead to a decline in infection rate and consequently a reduction in antibiotic consumption at the population level. In this empirical analysis, we estimated the potential impact of the IMI on private-sector antibiotic utilisation using a quasi-experimental approach and population-level antibiotic consumption data.

## Methods

### Data

We analysed the PharmaTrac dataset—a nationally representative drug sales audit dataset. The PharmaTrac sample covers 60% of stockists (intermediaries between carrying and forwarding agents and retail pharmacy shops in the pharmaceutical supply chain), which corresponds to 18,000 distributors, 32,000 sub-stockists, and 500,000 retailers spread across 23 different regions in India [14]. The data is collected by a market research company, All-Indian Origin Chemists and Distributors Limited (AIOCD) and has been previously used to estimate antibiotic consumption in India [3, 15], impact evaluation of Schedule H1 - antimicrobial sales regulation in India [16], and several other research studies [17, 18]. The PharmaTrac Dataset uses the European Pharmaceutical Market Research Association (EphMRA) approach to arrange medicines into the Anatomical Therapeutic Chemical (ATC) classification. We employed this classification to identify systemic antibacterials (classified under J1), their dosage forms, and their utilisation in the private sector in terms of monetary value (Indian National Rupee) and volume for selected Indian states. The primary target population of the IMI was children under the age of 5 years; hence, we measured the potential impact of IMI on pediatric oral antibiotic use. We considered oral liquid antibiotic consumption as a proxy for paediatric oral antibiotic use and included only liquid dosage forms of systemic antibacterials in our analysis.

### Intervention under study

The intervention - IMI - was implemented from October 2017 to January 2018. It was implemented in 121 districts (including 52 districts from the Northeastern states) and 17 urban areas across the country. We selected Bihar (16 districts), Madhya Pradesh (14 districts), Maharashtra (11 districts), Rajasthan (12 districts), Uttar Pradesh (60 districts) and the Northeastern (52 districts) States for this research as these states contributed the majority of districts selected for IMI implementation. The study period was from January 2015 to December 2019. The time period was selected to ensure a sufficient number of pre-intervention (33 months) and post-intervention (27 months) data points to compare antibiotic usage rates before and after the implementation of IMI.

### Outcome

The primary outcome measure was ‘antibiotics sales volume per month’– a proxy for the utilisation of antibiotics in the private sector. We report the percentage change (increase or decrease) in the sales volume of oral liquid antibiotics after the implementation of IMI, measured in standard units (SUs), which is the smallest formulation dose (one bottle of oral liquid). We stratified the outcome data by selected Indian states (Bihar, Madhya Pradesh, Rajasthan, Maharashtra Uttar Pradesh-East, Uttar Pradesh-West and Northeastern States) and by antibiotic classes (Chloramphenicol and combinations (J1B), Broad spectrum penicillin (J1C), Cephalosporins (J1D), Trimethoprim and similar formulations (J1E), Macrolides and similar types (J1F), Fluoroquinolones (J1G)).

### Research design

Interrupted time series (ITS) [19, 20] - a quasi-experimental research design was used to estimate the potential impact of IMI on private-sector antibiotic utilisation in India.

### Statistical analysis

We conducted interrupted time series analyses by fitting a Poisson regression model with the Newey–West standard errors [21] to account for autocorrelation and heterogeneity of variance, i.e. heteroskedasticity. The model included a time variable, a dummy intervention variable indicating the pre-IMI period and post-IMI period, and an interaction term between time and the intervention variable. This approach takes account of pre-intervention trends and allows estimation of the effect of the intervention at various timepoints by centring time at that timepoint.

Our regression equation was as follows:

$$\begin{array}{l}\:{y}_{t}={\beta\:}_{0}+{\beta\:}_{1}*{time}_{t}+{\beta\:}_{2}*{intervention}_{t}\\+{\beta\:}_{3}*{time\:after\:intervention}_{t}+{\epsilon\:}_{t}\end{array}$$

where Y_t_ is the ‘sales volume’ of antibiotics per month at time t, β_0_ estimates the baseline sales volume of antibiotics at the beginning of the time series, prior to the intervention; β_1_: estimates the **baseline trend**; β_2_: estimates the change in sales volume of antibiotics in the first month following the intervention; and β_3_: estimates the **change in trend** in the post- intervention segment.

We estimated the immediate level change (β_2)_, post-IMI implementation trend change (β_3)_ and post-IMI implementation trend. The level change (β_2_) represents the difference in antibiotic utilisation between the specified post-intervention time point and the pre-intervention regression line that is extrapolated to that same time point (counterfactual). The trend change (β_3_) represents an increase or decrease in the slope of a time series segment after the intervention compared with the pre-intervention trend. The post-IMI implementation trend was estimated by adding together the coefficients associated with time and the time-intervention interaction.

To account for seasonal changes in antibiotic utilisation, we included two pairs of sine and cosine terms (Fourier terms) in the model. The model was checked for autocorrelation with the help of autocorrelation (ac) and partial autocorrelation (pac) estimates and plots of the residuals, and appropriate adjustments were made to the model. The 95% confidence interval was generated using standard error (SE) that was estimated using the post-estimation command (after Poisson regression) which was then used to generate time series graphs. The analysis was carried out using the statistical software, STATA version 18.

## Results

**Fig. 1:**
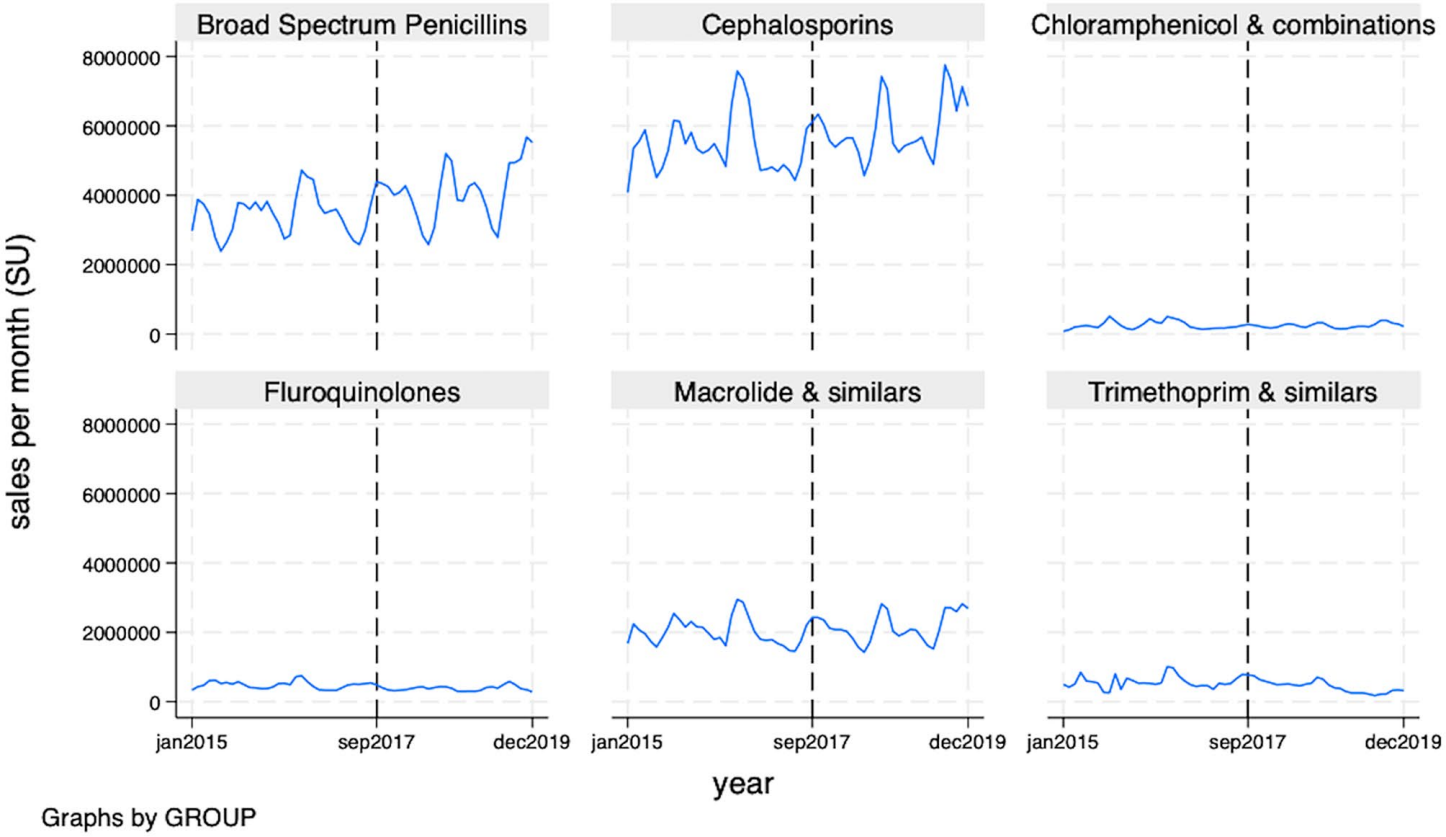
Private-sector monthly antibiotic sales across antibiotic class, before and after Intensified Mission Indradhanush implementation, January 2015–December 2019. Note: The vertical line on the x-axis represents the Intensified Mission Indradhanush introduction

Figure 1 (and Table S1 in Additional file 1) present the unadjusted median monthly sales of antibiotic classes before and after the implementation of IMI. In the 27 months after the implementation of IMI in October 2017 (post-intervention period), compared to 33 months before IMI implementation. All antibiotic classes had a higher median monthly sale except Fluoroquinolones and Trimethoprim. We also report unadjusted median monthly antibiotic sales disaggregated by selected Indian states (Table S2, Additional File 1). All states except the Northeastern states reported a higher median monthly sale in the 27 months after the implementation of IMI in October 2017 compared to 33 months before IMI implementation.

**Table 1:** Poisson segmented regression models of the impact of intensified mission Indradhanush on private-sector antibiotic utilisation in India

Antibiotics	Pre-IMI implementation intervention trend*	Post- IMI implementation level change	Post- IMI implementation trend change*	Post- IMI implementation trend*
Broad spectrum penicillin	1.002 (0.998- 1.005)	1.002 (0.906- 1.108)	1.007 (1.001- 1.012)	1.009 (1.004- 1.013)
Cephalosporins	0.999 (0.995- 1.002)	1.029 (0.933- 1.135)	1.006 (1.000- 1.011)	1.005 (1.000- 1.010)
Chloramphenicol and combinations	0.994 (0.985- 1.003)	0.994 (0.756–1.307)	1.010 (0.994- 1.026)	1.004 (0.991- 1.017)
Fluoroquinolones	0.994 (0.990–0.998)	0.874 (0.777–0.983)	1.006 (0.999- 1.013)	1.000 (0.995–1.006)
Macrolides and similar types	0.996 (0.992- 1.000)	1.030 (0.924–1.150)	1.010 (1.003–1.015)	1.005 (1.000- 1.010)
Trimethoprim and similar formulations	1.002 (0.993–1.011)	1.171 (0.901- 1.522)	0.957 (0.941- 0.973)	0.959 (0.945- 0.973)

Data are incidence rate ratio (95% CI) or trend (95% CI). *Slope change per month

**Fig. 2:**
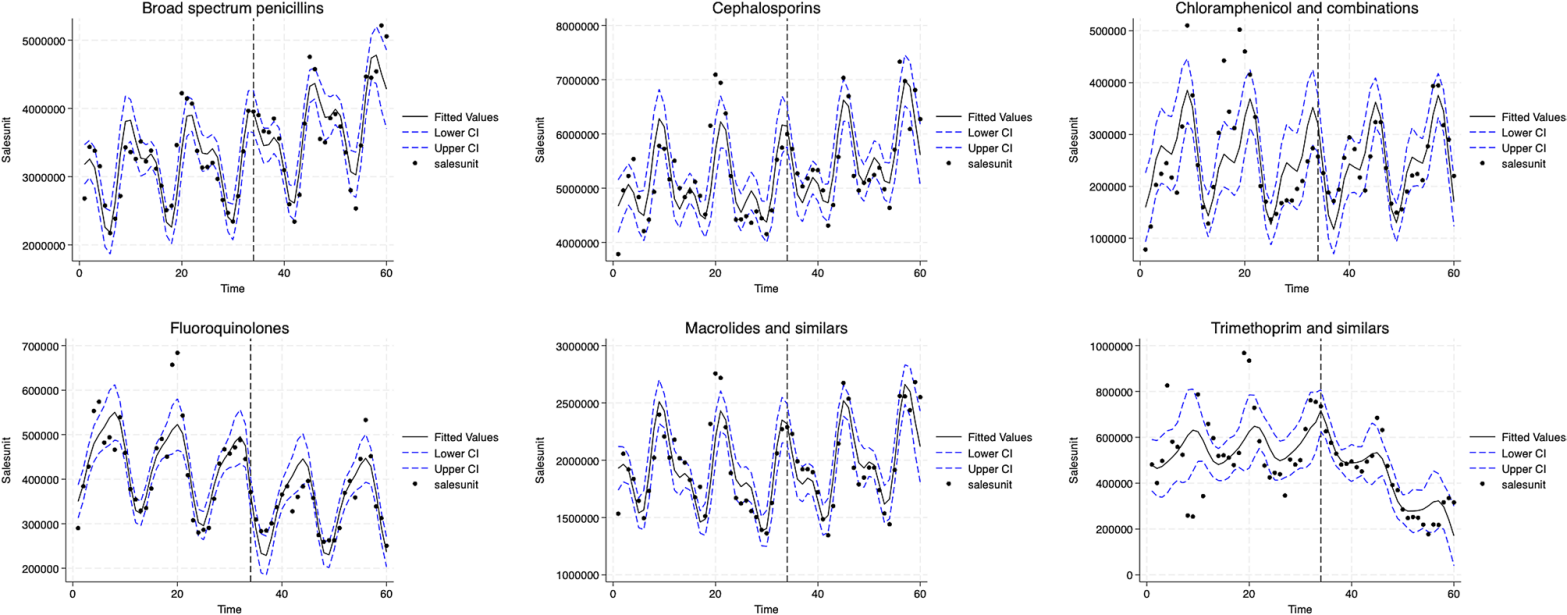
Poisson segmented regression models of the impact of Intensified Mission Indradhanush on private-sector antibiotic sales in India

Table 1; Fig. 2 present the potential impact of IMI on private-sector antibiotic utilisation. The Poisson segmented regression analysis showed a 12.6% decrease in Fluoroquinolones sales in the first month of IMI implementation (October 2017) (incidence rate ratio [IRR] 0.874, 95% CI (0.777–0.983). However, post-intervention, the fluoroquinolone sales remained broadly constant (IRR 1.000, 95% CI 0.995–1.006). Similarly, there was a 0.6% (IRR 0.994, 95% CI 0.756–1.307) decrease in Chloramphenicol sales in the first month; thereafter, the sales increased by a trend of 0.4% per month (IRR 1.004, 95% CI 0.991–1.017) in the post-intervention period. Interestingly, there was a 17.1% increase in Trimethoprim sales in the first month of IMI implementation, but the sales decreased by 0.4% per month (IRR 0.959, 95% CI 0.945–0.973) during the post-intervention period.

However, there was an increase in sales of Macrolides by 3.0% (IRR 1.030, 95% CI 0.924–1.150), Cephalosporins by 2.9% (IRR 1.029, 95% CI 0.933–1.135) and broad-spectrum Penicillin by 0.2% (IRR 1.002, 95% CI 0.906–1.108) in the first month of the IMI implementation. Thereafter, in the post-intervention period, Macrolides sales increased by a monthly rate of 0.5% (IRR 1.005, 95% CI 1.000–1.010), Cephalosporins sales increased by 0.5% per month (IRR 1.005, 95% CI 1.000 -1.010) and Broad spectrum penicillin sales increased by 0.9% per month (IRR 1.009, 95% CI 1.004–1.013). Since the sales of antibiotics may be influenced by the changes in the population, we conducted a sensitivity analysis using a Poisson regression model that accounts for population size by including log (Population) as an offset term. However, the results remain unchanged. (Table S3, Additional File 1)

**Table 2:** Poisson segmented regression models of the impact of intensified mission Indradhanush on private-sector antibiotic utilisation across Indian States

State	Pre-IMI implementation intervention trend*	Post- IMI implementation level change	Post- IMI implementation trend change*	Post- IMI implementation trend*
Bihar	0.999 (0.995–1.003)	0.959 (0.851- 1.081)	1.013 (1.006–1.020)	1.012 (1.007- 1.018)
Madhya Pradesh	0.993 (0.988 0.999)	1.161 (0.999–1.348)	1.011 (1.003- 1.020)	1.005 (0.998- 1.011)
Northeastern States	0.997 (0.993- 1.001)	0.977 (0.861–1.107)	0.997 (0.990–1.004)	0.994 (0.988-1.000)
Rajasthan	1.003 (0.999 1.006)	0.984 (0.878–1.102)	0.997 (0.990- 1.003)	0.999 (0.994–1.004)
Uttar Pradesh-East	0.999 (0.995- 1.005)	1.022 (0.887- 1.179)	1.003 (0.995 1.012)	1.003 (0.997- 1.010)
Uttar Pradesh-West^#^	1.001 (0.996- 1.005)	0.995 (0.873–1.134)	1.008 (1.000- 1.015)	1.009 (1.003- 1.015)
Maharashtra^##^	0.999 (0.994- 1.003)	1.095 (0.974- 1.233)	1.001 (0.994- 1.007)	0.999 (0.994- 1.005)

Reporting for Uttar Pradesh, as UP-east and UP-west, is driven by the data collection and reporting system adopted in the PharmaTrac dataset. ^#^UP-West also includes data from Uttarakhand, ^##^ data for Maharashtra excludes data from Mumbai region

Data are incidence rate ratio (95% CI) or trend (95% CI). *Slope change per month

Table 2 presents the potential impact of IMI on total antibiotic utilisation across Indian states. The segmented regression analysis showed weak evidence of a small decrease in antibiotic sales in the first month of IMI implementation (October 2017) in Northeastern States (IRR 0.977, 95% CI 0.861–1.107) and Rajasthan (IRR 0.984, 95% CI 0.878–1.102). Post-IMI implementation (after October 2017), antibiotic sales decreased by a trend of 0.6% per month (IRR 0.994, 95% CI 0.988-1.000) in Northeastern states and decreased by a rate of 0.1% per month (IRR 0.999, 95% CI 0.994–1.004) in Rajasthan. However, in Bihar and Uttar Pradesh-West, antibiotic sales showed a decrease in the first month of IMI implementation (October 2017) by 4.1% (IRR 0.959, 95% CI 0.851–1.081) and 0.5% (IRR 0.995, 95% CI 0.873–1.134), respectively. Thereafter, in the post-intervention period, antibiotic sales increased by a trend of 1.2% per month (IRR 1.012, 95% CI 1.007–1.018) in Bihar and by 0.9% per month (IRR 1.009, 95% CI 1.003–1.015) in Uttar Pradesh-West.

Furthermore, there was an increase in antibiotic sales in Madhya Pradesh by 16.1% (IRR 1.161 (0.999–1.348), in Maharashtra by 9.5% (IRR 1.095, 95% CI 0.974–1.233) and Uttar Pradesh-East by 2.2% (IRR 1.022 (0.887–1.179) in the first month of the IMI implementation which persisted in post-intervention period, at a monthly rate of 0.5% (IRR 1.005, 95% CI 0.998–1.011) in Madhya Pradesh, 0.3% per month (IRR 1.003, 95% CI 0.997–1.010) in Uttar Pradesh -East increased but remained broadly constant (IRR 0.999, 95% CI 0.994–1.005) in Maharashtra.

There was heterogeneity in antibiotic utilisation by the antibiotic class type (Table S4, Figures S1-S5 in Additional File 1) across Indian states, which was driving the country-level IMI impact for each antibiotic class under consideration. For example, only Trimethoprim sales showed a decreasing post-intervention trend relative to the pre-intervention trend across all States. Broad-spectrum Penicillins, Cephalosporins and Fluoroquinolones had a decreasing post-intervention trend in Rajasthan, while Macrolides showed an increasing post-intervention trend across all States except the Northeastern states.

## Discussion

We present the potential impact of periodic intensification of routine immunisation strategy - IMI - on private-sector antibiotic utilisation in India. Our results suggest a decrease in the monthly sales of Trimethoprim and broadly constant sales of Fluoroquinolones in the post-intervention period. However, there was a minor increase in monthly antibiotic sales (range 0.5 − 0.9% per month) post-IMI implementation for broad-spectrum Penicillins, Cephalosporins, and Macrolides. Furthermore, there was heterogeneity in the impact of IMI on total antibiotic utilisation across Indian states. In the post-IMI implementation period, Rajasthan and Northeastern states had a decrease in antibiotic sales while Bihar, Madhya Pradesh and Uttar Pradesh had an increase.

Several studies have estimated the potential impact of IMI from different perspectives, some estimated the impact of IMI on full immunisation coverage while others on an increase in antigen-specific vaccine delivery rate. Gurnani et al. reported that full immunisation coverage in IMI districts increased by 18.5% from pre-IMI levels [12]. Similarly, using a controlled interrupted time series approach, Clarke-Deelder et al. analysed district-level data and reported that IMI resulted in increased delivery of traditional UIP vaccines, with a median effect of 10.6% (95% CI 5.1–16.5%) during the implementation period [13]. However, none of these recently published studies analysed the relationship between immunisation and demand for antibiotics. Ours is the first and only study that has attempted to quantify the potential impact of IMI on antibiotic utilisation, particularly oral liquid antibiotic preparations, which are predominantly used in children. Private-sector antibiotic utilisation is a good proxy for actual antibiotic use at the community level in India since 85–90% of medicine expenditure is incurred in the private sector [22]. 

Recent research on the effects of vaccination on antibiotic consumption has also confirmed that the pneumococcal vaccine provides 19.7% (95% CI, 3.4–43.4%) protection and the rotavirus vaccine confers 11.4% (4.0–18.6%) protection against antibiotic-treated episodes of acute respiratory infection and diarrhoea, respectively [23]. A meta-analysis also confirmed that pneumococcal vaccination (Ratio of Means 0.92, 95% CI 0.85–1.00) and influenza vaccination (Ratio of Means 0.71, 95% CI 0.62–0.83) results in a decrease in the number of antibiotic prescriptions [24]. However, there is a knowledge gap in the dynamic relationship between the intensification of routine immunisation activities and the demand for antibiotics at the population level. Our study attempts to answer this question. We observed heterogeneity in the trend of antibiotic utilisation in the post-IMI implementation period. One of the possible reasons for no significant decline in the post-intervention trend of broad-spectrum Penicillins, Cephalosporins and Macrolides is their broad spectrum of activity and indications. These antibiotics are widely prescribed for respiratory tract infections, the majority of which are viral in origin [2]. Hence, the likelihood of overuse of these antibiotics is high along with classical respiratory antibiotics such as Macrolides. Increased use of these antibiotics may have influenced the reduction in Trimethoprim utilisation post-IMI implementation.

Another possible explanation is the substitutability of antibiotics [2] across indications which makes it easier for prescribers to use broad-spectrum antibiotics, especially in the private sector. Previous research has indicated that though the per-capita antibiotic consumption rate in India is relatively low compared to many countries, consumption of broad-spectrum antibiotics remains high [2, 15, 18]. A reduction in fluoroquinolones may be a reflection of a reduction in diarrhoeal diseases at the population level because of the introduction of the rotavirus vaccine in the immunisation program [25]. Another possible reason is their substitutability with cephalosporins for several indications resulting in lower prescriptions, as they are not recommended in children aged less than 5 years [26]. 

We also observed wide heterogeneities for the impact of the IMI across states. The declining trend in state-level antibiotic utilisation per month post-IMI implementation in the Northeastern states is a reflection of the intensive nature of the IMI implementation in the Northeastern states. As per the operational guidelines, among the 190 districts selected for the IMI, 57 (30%) were from Northeastern states. Furthermore, most districts in the northeast recorded more than a 20% point increase in full immunisation coverage between the National Family Health Survey-4 and Intensive Mission Indradhanush round [27]. They also reported more than 75% coverage for key antigens [27]. Hence, the potential impact of IMI on reducing the demand for antibiotics is comparatively higher in Northeastern states compared to other states.

Though several districts in Uttar Pradesh, Madhya Pradesh, Bihar and Maharashtra had reported more than a 20% increase in full immunisation coverage there was no significant reduction in state-level antibiotic utilisation in the post-IMI implementation period. One of the reasons could be the easy availability of antibiotics over the counter at retail pharmacies which may have resulted in the overuse of antibiotics [28–30] that might have blunted the impact of IMI. Another explanation may be heterogeneity in the intensity of IMI implementation and hence immunisation coverage rates– the marginal impact of IMI on demand for antibiotics is conditional on the immunisation coverage rates. Clarke-Deelder et al. also reported that there was no evidence of a sustained effect of IMI in the intervention districts, 8-months post-IMI implementation [13]. Furthermore, though antibiotics are prescribed for all types of infections, some of them may not be vaccine-preventable. Hence, demand for antibiotics may or may not reduce at the population level. For example, vaccines that prevent pneumonia, such as Pentavalent and PCV, and diarrhoea, such as the rotavirus vaccine, may have a limited impact on demand for antibiotics, as viral infections and other causes of pneumonia and diarrhoea would still result in antibiotic prescriptions and overuse.

For the states of Bihar, Madhya Pradesh, and Uttar Pradesh, one of the reasons for no significant reduction in antibiotic use could be high infection rates and hence higher rates of antibiotic prescription. Previous research has indicated these states have higher than the national mean all-age DALY rates for infectious disease conditions such as lower respiratory infections, tuberculosis, diarrhoeal diseases, and intestinal infections [1]. Furthermore, there is a delay in achieving a desired level of immunisation coverage that is commensurate with population-level herd immunity to protect non-vaccinated individuals. Recent research has also demonstrated that private antibiotic consumption was positively associated with private vaccine consumption when monthly lags were fewer than 18 months, but had a negative association when 32 or more months had elapsed after vaccine consumption [7].

The PharmaTrac data set has been used previously to conduct an impact evaluation of antimicrobial stewardship policies [16, 18]. As per the national health accounts estimates for India [22], around 85–90% of medicine expenditure is incurred in the private sector. Hence, our impact IMI estimates are likely to capture the true effect size. However, our study has some limitations. First, we could not estimate the potential impact of IMI on public-sector antibiotic use as PharmaTrac data does not capture public-sector antibiotic use. Second, since the PharmaTrac data is aggregated at the state level, we could not report district-level estimates and impact. Third, antibiotic sales data do not capture the actual antibiotic consumption by an individual; hence, age and gender-specific outcomes could not be estimated.

## Conclusion

We found heterogeneity in the impact of IMI implementation on private-sector antibiotic utilisation across antibiotic classes as well as the Indian states. Though we used nationally representative data, the estimated effect sizes were small. We recommend that the impact evaluation of immunisation on antibiotic utilisation should be carried out using population-based cohorts not only for new vaccine introductions but also for periodic intensification of routine immunisation strategies.

## Electronic supplementary material

Below is the link to the electronic supplementary material.

**Additional File 1**: **Table S1**. Summary statistics of private sector antibiotic sales, before and after Intensified Mission Indradhanush implementation in India, January 2015–December 2019. **Table S2**. Summary statistics of private sector antibiotic sales, before and after Intensified Mission Indradhanush implementation across Indian states, January 2015–December 2019. **Table S3**. Poisson segmented regression models of the impact of Intensified Mission Indradhanush on private-sector antibiotic utilisation across antibiotic classes, with population size included as an offset term (log-transformed). **Table S4**. Poisson segmented regression models of the impact of Intensified Mission Indradhanush on private-sector antibiotic utilisation across antibiotic classes and Indian States. **Figure S1**. Poisson segmented regression models of the impact of Intensified Mission Indradhanush on private-sector antibiotic sales in Bihar. **Figure S2**. Poisson segmented regression models of the impact of Intensified Mission Indradhanush on private-sector antibiotic sales in Madhya Pradesh. **Figure S3**. Poisson segmented regression models of the impact of Intensified Mission Indradhanush on private-sector antibiotic sales in the Northeastern States. **Figure S4**. Poisson segmented regression models of the impact of Intensified Mission Indradhanush on private-sector antibiotic sales in Rajasthan. **Figure S5**. Poisson segmented regression models of the impact of Intensified Mission Indradhanush on private-sector antibiotic sales in UP.

## Data Availability

The data that support the findings of this study are available from PharmaTrac but restrictions apply to the availability of these data. (https://www.aiocdawacs.com/(S(closk2ozinyrosjywwc21yxf))/ProductDetail.aspx)

## Abbreviations

AICOD: All-Indian Origin Chemists and Distributors Limited
ATC: Anatomical Therapeutic Chemical
AMR: Antimicrobial Resistance
DALY: Disability Adjusted Life Years
DRI: Drug Resistance Index
EphMRA: European Pharmaceutical Market Research Association
GBD: Global Burden of Disease
IMI: Intensified Mission Indradhanush
ITS: Interrupted time series
IRR: Incidence Risk Ratio
MI: Mission Indradhanush
NFHS: National Family Health Survey
UIP: Universal Immunisation Program
