# Supplementary material for: Did the periodic intensification of routine immunisation strategy (Intensified mission Indradhanush) reduce the demand for pediatric antibiotic formulations in India?
Source: BMC Infect Dis. 2025 May 24;25:741. doi: 10.1186/s12879-025-11082-3 (PMC12102883; doi:10.1186/s12879-025-11082-3)
Supplement: Supplementary file 1 — Additional File 1: Table S1. Summary statistics of private sector antibiotic sales, before and after Intensified Mission Indradhanush implementation in India, January 2015–December 2019. Table S2. Summary statistics of private sector antibiotic sales, before and after Intensified Mission Indradhanush implementation across Indian states, January 2015–December 2019. Table S3. Poisson segmented regression models of the impact of Intensified Mission Indradhanush on private-sector antibiotic utilisation across antibiotic classes, with population size included as an offset term (log-transformed). Table S4. Poisson segmented regression models of the impact of Intensified Mission Indradhanush on private-sector antibiotic utilisation across antibiotic classes and Indian States. Figure S1. Poisson segmented regression models of the impact of Intensified Mission Indradhanush on private-sector antibiotic sales in Bihar. Figure S2. Poisson segmented regression models of the impact of Intensified Mission Indradhanush on private-sector antibiotic sales in Madhya Pradesh. Figure S3. Poisson segmented regression models of the impact of Intensified Mission Indradhanush on private-sector antibiotic sales in the Northeastern States. Figure S4. Poisson segmented regression models of the impact of Intensified Mission Indradhanush on private-sector antibiotic sales in Rajasthan. Figure S5. Poisson segmented regression models of the impact of Intensified Mission Indradhanush on private-sector antibiotic sales in UP. [file 12879_2025_11082_MOESM1_ESM.docx]

**Additional File 1**

Table S1. Summary statistics of private sector antibiotic sales, before and after Intensified Mission Indradhanush implementation in India, January 2015–December 2019

| Antibiotics | Pre-IMI implementation  (sales per month, median (IQR)) | Post- IMI implementation  (sales per month, median (IQR)) |
| --- | --- | --- |
| Broad spectrum penicillins | 3152,976 (2682,102 3382,591) | 3736,350 (3356,310 4450,793) |
| Cephalosporins | 4937,484 (4484,442 5526,366) | 5331,091 (5032,794 6089,435) |
| Chloramphenicol and combinations | 216,976 (172,635 315,332) | 225,563 (191,956 290,191) |
| Fluoroquinolones | 450,676 (356,026 487,890) | 338,961 (284,575 383,701) |
| Macrolides and similar types | 1836,849 (1627,789 2060,552) | 1933,440 (1738,531 2436,871) |
| Trimethoprim and similar formulations | 519,186 (476,626 635,896) | 451,032 (252,341 519,364) |

Number of observations: pre-intervention: 33 and post-intervention: 27

Table S2. Summary statistics of private sector antibiotic sales, before and after Intensified Mission Indradhanush implementation in India, January 2015–December 2019

| State | Pre-intervention (sales per month, median (IQR)) | Post-intervention  (sales per month, median (IQR) |
| --- | --- | --- |
| Bihar | 2199,593 (2038,765 2452332) | 2610,096 (2275369 2957062) |
| MP | 875,230 (711580 1128143) | 1018,076 (859177 1159098) |
| North East States | 1250,205 (1098619 1403778) | 1040,966 (902822 1133982) |
| Rajasthan | 1123,596 (989872 1221101) | 1262,275 (1039590 1372807) |
| UP East | 1479,695 (1351018 1805652) | 1624,244 (1530551 2010293) |
| UP West | 1784,096 (1471237 1998359) | 1962,160 (1829609 2463884) |
| Maharashtra | 1702,523 (1558160 1896308) | 1875,396 (1614542 2173829) |

Number of observations: pre-intervention: 33 and post-intervention: 27

Reporting for Uttar Pradesh as UP-east and UP-west is driven by the data collection and reporting system adopted in the PharmaTrac dataset. ^#^UP-West also includes data from Uttarakhand, ^##^ data for Maharashtra excludes data from Mumbai region

Table S3. Poisson segmented regression models of the impact of Intensified Mission Indradhanush on private-sector antibiotic utilisation across antibiotic classes, with population size included as an offset term (log-transformed)

| Antibiotics | Offset: log(Population) | Intercept | Pre-IMI implementation intervention trend* | Post- IMI implementation level change | Post- IMI implementation trend change* | Post- IMI implementation trend* |
| --- | --- | --- | --- | --- | --- | --- |
| Broad spectrum penicillin | 1 (offset) | 24.6 | 1.002 (0.998- 1.005) | 1.002 (0.906- 1.108) | 1.007 (1.001- 1.012) | 1.009 (1.004- 1.013) |
| Cephalosporins | 1 (offset) | 42.2 | 0.999 (0.995- 1.002) | 1.029 (0.933- 1.135) | 1.006 (1.000- 1.011) | 1.005 (1.000- 1.009) |
| Chloramphenicol and combinations | 1 (offset) | 2.19 | 0.994 (0.985- 1.003) | 0.994 (0.756-1.307) | 1.010 (0.994- 1.026) | 1.004 (0.991- 1.017) |
| Fluoroquinolones | 1 (offset) | 3.80 | 0.994 (0.990-0.998) | 0.874 (0.777- 0.983) | 1.006 (0.999- 1.013) | 1.000 (0.995-1.006) |
| Macrolides and similar types | 1 (offset) | 16.4 | 0.996 (0.992- 1.000) | 1.030 (0.924-1.150) | 1.009 (1.003-1.015) | 1.005 (1.000- 1.010) |
| Trimethoprim and similar formulations | 1 (offset) | 4.35 | 1.002 (0.993- 1.011) | 1.171 (0.901- 1.522) | 0.957 (0.941- 0.973) | 0.959 (0.945- 0.973) |

Note: model accounts for population size by including log(Population) as an offset, which adjusts the counts to rates; Data are incidence rate ratio (95% CI) or trend (95% CI); *Slope change per month

Table S4. Poisson segmented regression models of the impact of Intensified Mission Indradhanush on private-sector antibiotic utilisation across antibiotic classes and Indian States

| State | Antibiotic class | Pre-IMI implementation intervention trend* | Post- IMI implementation level change | Post- IMI implementation trend change* |
| --- | --- | --- | --- | --- |
| Bihar | Broad spectrum penicillins | 1.002 (1.000- 1.004) | 0.893 (0.850- 0.938) | 1.021 (1.018-1.023) |
|  | Cephalosporins | 0.997 (0.994-1.000) | 1.069 (1.004-1.138) | 1.012 (1.009- 1.016) |
|  | Chloramphenicol and combinations | 1.010 (0.999- 1.021) | 0.851 (0.716- 1.011) | 0.996 (0.983- 1.010) |
|  | Fluoroquinolones | 0.983 (0.980-0.986) | 0.354(0.260- 0.480) | 1.043 (1.026- 1.060) |
|  | Macrolides and similar types | 0.998 (0.995- 1.001) | 0.910 (0.794- 1.043) | 1.017 (1.010-1.0256) |
|  | Trimethoprim and similar formulations | 1.005 (0.998- 1.012) | 1.250 (1.056- 1.480) | 0.919 (0.909- 0.929) |
| Madhya Pradesh | Broad spectrum penicillins | 0.999 (0.998-1.001) | 1.103 (1.041-1.167) | 1.011 (1.008- 1.014) |
|  | Cephalosporins | 0.989 (0.987- 0.992) | 1.212 (1.149-1.278) | 1.016 (1.013-1.020) |
|  | Chloramphenicol and combinations | 1.004 (0.993-1.016) | 0.896 (0.715- 1.123) | 1.016 (1.002-1.030) |
|  | Fluoroquinolones | 0.989 (0.987- 0.990) | 0.915 (0.839- 0.998) | 1.027 (1.022- 1.032) |
|  | Macrolides and similar types | 0.993 (0.990-0.995) | 1.206 (1.136- 1.280) | 1.012 (1.010-1.015) |
|  | Trimethoprim and similar formulations | 1.000 (0.996-1.003) | 1.260 (1.107-1.434) | 0.943 (0.935- 0 .952) |
| North East States | Broad spectrum penicillins | 0.999 (0.996- 1.002) | 0.898 (0.837- 0963) | 1.001 (0.996-1.005) |
|  | Cephalosporins | 0.995 (0.993- 0.998) | 1.007 (0.950- 1.067) | 1.000 (0.997- 1.003) |
|  | Chloramphenicol and combinations | 0.982 (0.975- 0.988) | 1.85 (1.530-2.23) | 1.013 (1.004- 1.022) |
|  | Fluoroquinolones | 1.002 (1.001- 1.003) | 0.783 (0.746- 0.822) | 1.001 (0.998- 1.005) |
|  | Macrolides and similar types | 0.997 (0.993- 1.002) | 1.083 (0.990- 1.186) | 0.987 (0.981-0.992) |
|  | Trimethoprim and similar formulations |  |  |  |
| Rajasthan | Broad spectrum penicillins | 1.006 (1.000-1.012) | 0.987 (0.868-1.123) | 0.997 (0.989- 1.005) |
|  | Cephalosporins | 1.002 (0.997-1.006) | 0.988 (0.877-1.114 | 0.993 (0.987-0.999) |
|  | Chloramphenicol and combinations | 1.001 (0.988- 1.014) | 0.601 (0.397- 0.910 | 1.009 (0.986- 1.032) |
|  | Fluoroquinolones | 1.004 (0.999-1.009) | 0.908 (0.795- 1.039) | 0.997 (0.989-1.006) |
|  | Macrolides and similar types | 0.996 (0.991- 1.001) | 0.942 (0.819- 1.0838) | 1.012 (1.005- 1.019) |
|  | Trimethoprim and similar formulations | 1.006 (0.991- 1.022) | 1.623 (1.061-2.483) | 0.926 (0.895 -0.958) |
| UP East | Broad spectrum penicillins | 0.996 (0.994-0.998) | 1.090 (1.034-1.149) | 1.011 (1.009- 1.014) |
|  | Cephalosporins | 1.004 (0.999- 1.010) | 0.979 (0.891-1.077) | 1.001 (0.995-1.007) |
|  | Chloramphenicol and combinations | 0.990 (0.985- 0.995) | 1.512 (1.303- 1.756) | 1.009 (1.003- 1.016) |
|  | Fluoroquinolones | 0.999 (0.997- 1.000) | 1.055 (0.984- 1.131) | 0.983 (0.979- 0.987) |
|  | Macrolides and similar types | 0.987 (0.981- 0.992) | 1.093 (0.980- 1.219) | 1.019 (1.013- 1.025) |
|  | Trimethoprim and similar formulations | 1.003 (0.994- 1.011) | 1.136 (0.946- 1.366 | 0.929 (0.919- 0.939) |
| UP West | Broad spectrum penicillins | 1.004 (1.000- 1.008) | 1.016 (0.934-1.104) | 1.002 (0.998 - 1.006) |
|  | Cephalosporins | 1.002 (0.997- 1.006) | 0.943 (0.865- 1.028) | 1.013 (1.009-1.0176) |
|  | Chloramphenicol and combinations | 0.978 (0.962- 0.995) | 1.053 (0.750-1.478) | 1.017 (0.999-1.036) |
|  | Fluoroquinolones | 0.993 (0.989- 0.996) | 0.963 (0.898- 1.033) | 1.008 (1.005- 1.012) |
|  | Macrolides and similar types | 1.000 (0.995- 1.004) | 1.012 (0.919- 1.115) | 1.001 (0.997- 1.006) |
|  | Trimethoprim and similar formulations | 1.007 (1.001- 1.012) | 1.167 (1.047-1.301) | 0.996 (0.990- 1.002) |
| Maharashtra | Broad spectrum penicillins | 1.002 (1.001- 1.003) | 1.108 (1.020- 1.203) | 1.001 (0.996- 1.005) |
|  | Cephalosporins | 0.997 (0.996-0.998) | 1.095 (1.037- 1.158) | 0.998 (0.995-1.002) |
|  | Chloramphenicol and combinations | 0.990 (0.987- 0.994) | 1.197 (1.107- 1.295) | 1.016 (1.012- 1.020) |
|  | Fluoroquinolones | 0.995 (0.993- 0.996) | 0.951 (0.918- 0.985) | 1.003 (1.001-1.005) |
|  | Macrolides and similar types | 0.997 (0.995-0.999) | 1.097 (1.031- 1.167) | 1.005 (1.001- 1.008) |
|  | Trimethoprim and similar formulations | 0.997 (0.995- 0.999) | 1.282 (1.172- 1.401) | 0.943 (0.935- 0.952) |

Reporting for Uttar Pradesh as UP-east and UP-west is driven by the data collection and reporting system adopted in the PharmaTrac dataset. ^#^UP-West also includes data from Uttarakhand, ^##^ data for Maharashtra excludes data from Mumbai region; Data are incidence rate ratio (95% CI) or trend (95% CI). *Slope change per month

Table S5. National Immunisation Schedule
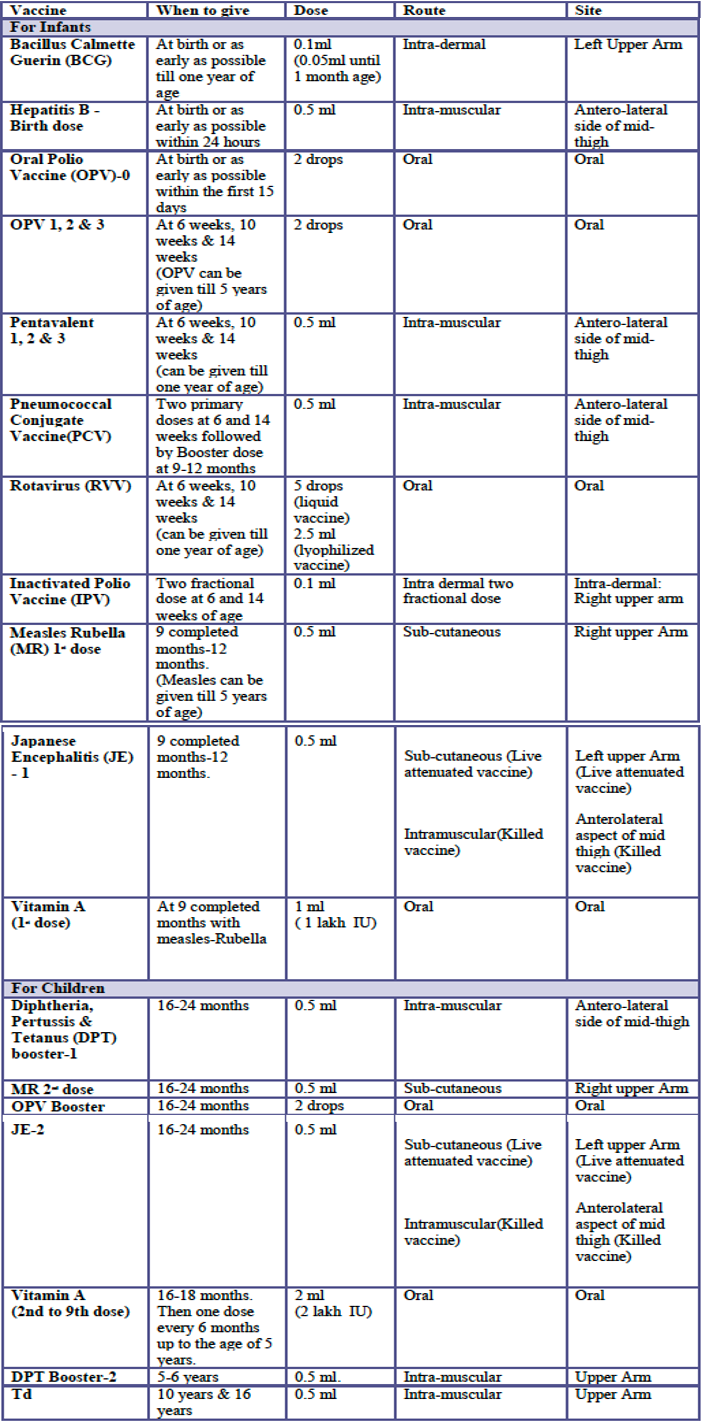

Figure S1. Poisson segmented regression models of the impact of Intensified Mission Indradhanush on private-sector antibiotic sales in Bihar

Figure S2. Poisson segmented regression models of the impact of Intensified Mission Indradhanush on private-sector antibiotic sales in Madhya Pradesh

Figure S3. Poisson segmented regression models of the impact of Intensified Mission Indradhanush on private-sector antibiotic sales in the Northeastern States

Figure S4. Poisson segmented regression models of the impact of Intensified Mission Indradhanush on private-sector antibiotic sales in Rajasthan

Figure S5. Poisson segmented regression models of the impact of Intensified Mission Indradhanush on private-sector antibiotic sales in UP
